# Case report: Minimally invasive primary debulking surgery for advanced stage epithelial ovarian cancer

**DOI:** 10.3389/fonc.2024.1302724

**Published:** 2024-02-02

**Authors:** Jennifer Wolf, Nicole Goncalves, Ioannis Alagkiozidis

**Affiliations:** ^1^ State University of New York (SUNY) Downstate Health Sciences University, Department of Obstetrics & Gynecology, Brooklyn, NY, United States; ^2^ Maimonides Medical Center, Department of Obstetrics & Gynecology, Brooklyn, NY, United States; ^3^ Maimonides Medical Center, Department of Gynecologic Oncology, Brooklyn, NY, United States

**Keywords:** ovarian cancer, minimally invasive surgery, robotic-assisted surgery, debulking, cytoreductive surgery

## Abstract

The surgical management of advanced ovarian cancer has historically emphasized an open technique, but advances in minimally invasive surgery (MIS) have led to its increasing use in ovarian cancer. Most research has focused on the utility of MIS in the interval debulking setting. Here, we present a case of a 38-year-old patient with incidentally diagnosed advanced stage ovarian cancer. We describe the robotic surgery techniques used to achieve complete primary cytoreduction, including resection of disease on the diaphragm. The patient has completed standard adjuvant chemotherapy and maintenance treatment and remains without evidence of disease for more than 2 years. This case details the techniques utilized to achieve complete cytoreduction including trocar placement, robotic instrument preference, and rotation of the robotic boom. This patient has had successful perioperative and oncologic outcomes, and her case highlights the role for minimally invasive primary debulking surgery for select patients with advanced ovarian cancer.

## 1 Introduction

In the United States, ovarian cancer affects nearly 22,000 patients and results in 14,000 deaths annually (1). At the time of diagnosis, approximately 60% of cases present with distant metastasis (1, 2). The primary modes of treatment revolve around surgery and chemotherapy, with a strong focus on achieving complete debulking to eliminate gross residual disease for the best possible outcomes. This objective can be achieved through primary surgery or interval debulking surgery (IDS) following neoadjuvant chemotherapy (3–6).

The integration of minimally invasive surgery (MIS) has introduced several advantages, including reduced hospital stay, diminished postoperative pain, and faster initiation of chemotherapy. However, it also introduces potential risks such as the rupture of an ovarian mass capsule, port site metastases, intraperitoneal tumor cell dissemination due to CO_2_ pneumoperitoneum, and incomplete debulking attributed to the lack of tactile feedback and suboptimal exposure. The diagnosis of ovarian cancer often occurs intraoperatively during laparoscopy. At this point, surgeons must decide whether to proceed immediately with staging via laparoscopy, convert to laparotomy, or schedule another procedure. Factors influencing this decision may include the need for counseling on staging procedures, discussions regarding fertility preservation, or referrals to specialized Gynecologic Oncology centers.

A comprehensive retrospective study comparing immediate laparoscopic primary staging for apparent early-stage disease to delayed surgery showed similar disease-free and overall survival rates. However, the immediate laparoscopic staging group had significantly higher rates of upstaging (25% vs 7%), ovarian capsule rupture (12% vs 2%), and conversion to laparotomy (16% vs 2%) (7). Small prospective feasibility trials examining MIS in the IDS setting have demonstrated its safety and similar recurrence rates compared to traditional open approaches (8, 9). In a large retrospective database study, patients undergoing IDS with MIS had lower 30- and 90-day mortality rates without adversely affecting overall survival (10). While most of the data comes from retrospective reviews or small prospective observational studies, an ongoing prospective randomized trial, the LANCE trial, is currently assessing the utility of MIS in the IDS setting. Similarly, in cases of recurrent disease, MIS has proven feasible in well-selected patient populations (11, 12).

Nevertheless, when it comes to primary debulking in advanced ovarian cancer, there is limited available data regarding the outcomes of MIS. Reports on the use of MIS for primary cytoreduction surgery are fewer compared to interval debulking. In small retrospective series, with careful patient selection, this approach appears to be feasible (13, 14). However, there is a lack of data with regard to the oncologic outcome and long term follow up with MIS in the setting of primary debulking for ovarian cancer.

Addressing some of the challenges associated with MIS in ovarian cancer, such as the need to operate in both the deep pelvis and upper abdomen and the technical difficulties posed by varying angles of operation, robotically-assisted MIS offers solutions. This technology allows for instrument undocking and boom rotation, along with increased degrees of motion provided by robotic joints and wristed instrument design.

**Figure 1:**
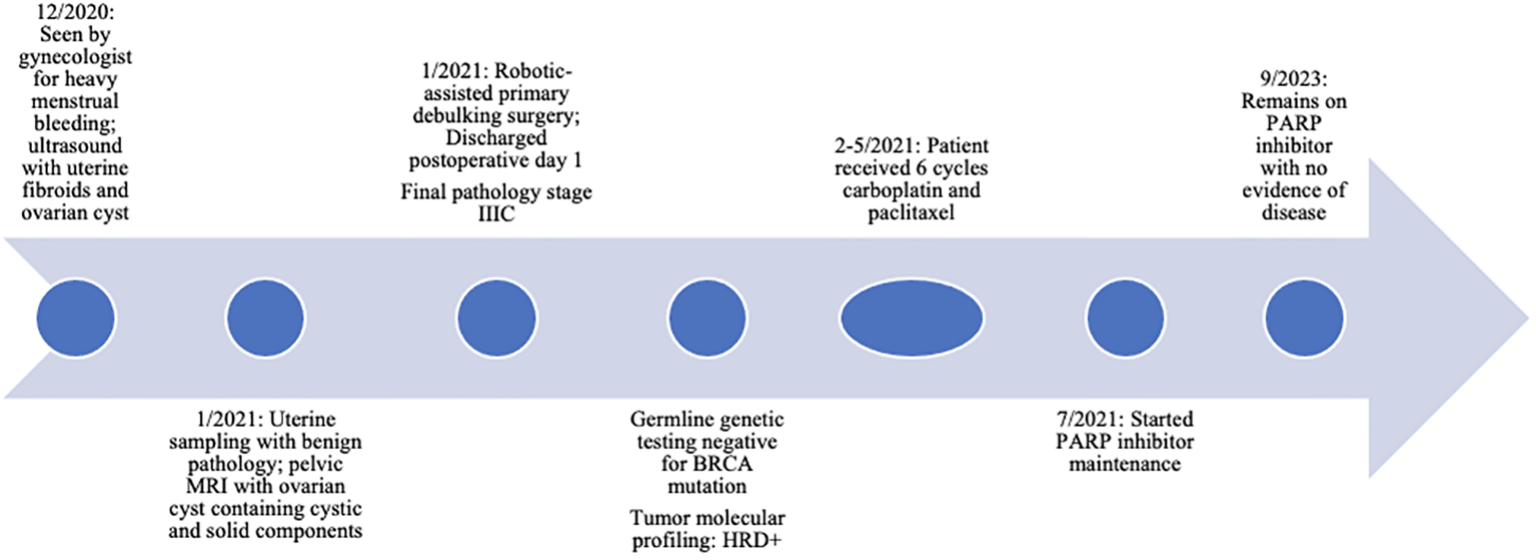
Clinical timeline of events. HRD, homologous recombination deficiency; PARP, poly (ADP-ribose) polymerase.

In this report, we present a case of advanced-stage ovarian cancer incidentally diagnosed during a robotic-assisted surgery. This case was optimally cytoreduced using a robotic-assisted minimally invasive technique, and the patient remains alive without evidence of disease more than 2 years later (**Figure 1**).

## 2 Case presentation

### 2.1 Presentation and surgical intervention

A 38-year-old woman, G1P1001, had a medical history of endometriosis, fibroids, and a left ovarian cyst. She had been under the care of her benign gynecologist due to heavy menstrual bleeding and new intermenstrual bleeding. She had previously been diagnosed with endometriosis after a laparoscopy 12 years ago and was also monitored for an ovarian cyst. Preoperative assessment included a pelvic ultrasound, which revealed uterine fibroids and a 4.6cm left ovarian cyst. A pelvic MRI confirmed adenomyosis and fibroids in addition to the left ovarian cyst, which measured 4.5cm and exhibited both solid and cystic components. The CA-125 level was 43. This cyst was thought to be related to her history of endometriosis and therefore no modeling score [such as an ADNEX model (15)] was employed. Endometrial sampling yielded benign pathology. She expressed her desire for surgical intervention to address her abnormal uterine bleeding. Consequently, her gynecologist planned to perform a robotic-assisted laparoscopic myomectomy and ovarian cystectomy, with the possibility of hysterectomy discussed depending on the intraoperative findings.

In the operating room, after the induction of general anesthesia, a supraumbilical 12 mm Hasson trocar was inserted using an open technique to insufflate the abdomen. Subsequently, four 8 mm robotic trocars were placed, two on each side, and an additional robotic trocar was inserted through the 12mm trocar, servicing as the camera port. Upon laparoscopic examination, a polypoid nodular implant was identified on the posterior fundal aspect of the uterus. Pelvic washings were collected, the robot was docked, and the implant was excised and sent for frozen section analysis. The results indicated a high-grade adenocarcinoma, prompting an intraoperative consultation with the Gynecologic Oncology team.

A comprehensive laparoscopic abdominal and pelvic examination revealed discrete pelvic nodules at the vesicouterine peritoneum, left pelvic sidewall, sigmoid colon, and descending colon, as well as two small right diaphragmatic nodules. A Fagotti score (16) was calculated as zero, indicating good probability of complete resection. There had not been preoperative imaging of the upper abdomen and chest, and therefore a Peritoneal Cancer Index (PCI) score (17) could not be calculated. However, all of the identified lesions appeared resectable, leading to the decision to proceed with robotic cytoreduction. The patient underwent a robotic-assisted total laparoscopic hysterectomy, bilateral salpingo-oophorectomy, pelvic and paraaortic lymph node dissection, infracolic omentectomy, and resection of nodules on the bladder serosa, pelvic peritoneum, and colon. A serosal defect on the sigmoid colon was repaired using 2-0 Quill sutures (Video 1). Subsequently, the robotic instruments were removed, the robotic platform was rotated 180 degrees, and the robotic was re-docked with the target anatomy at the diaphragm. The previously visualized diaphragmatic nodules, measuring approximately 1 cm and 5 mm, were resected, and a defect in the diaphragm was sutured using a 2-0 Quill suture in a running fashion (Video 2). Given the small size of the defect, a chest tube was not deemed necessary. Complete gross resection (R0) was successfully achieved, with an estimated blood loss of 50 cc and an operative time of 3 hours. The patient was admitted for overnight observation, experienced an uncomplicated postoperative course, and was discharged on postoperative day 1. Final pathology confirmed stage IIIC high-grade serous carcinoma of the ovary, with all excised nodules testing positive, including a 3 cm colonic lesion and the diaphragmatic nodules. The tumor cells expressed PAX-8, ER, CK7, and p16 and exhibited absent staining for p53.

### 2.2 Adjuvant therapy and surveillance

The patient completed 6 cycles of adjuvant chemotherapy with IV carboplatin and paclitaxel. This was initiated three weeks postoperatively and therapy was completed without delays. She had undergone genetic testing and tumor molecular profiling, which was negative for a BRCA mutation but positive for homologous recombination deficiency (HRD). After completing chemotherapy, CT of the chest, abdomen, and pelvis confirmed no evidence of disease, and she was started on a PARP inhibitor for maintenance therapy. This was dose-reduced due to anemia, but she has otherwise tolerated this well.

She continues to follow for surveillance visits with no clinical signs or symptoms of recurrence, and her CA-125 has remained low, 12-16. At the time of publication, she remains without evidence of disease for 27 months since completing adjuvant chemotherapy (**Figure 1**).

## 3 Discussion

The NCCN guidelines on ovarian cancer surgery principles recommend that most patients undergo laparotomy via a midline vertical incision for primary debulking, interval debulking, or secondary cytoreduction. Laparoscopy is considered valuable in assessing the feasibility of a cytoreductive procedure and in selected cases of interval debulking or early-stage disease (18). However, the field of gynecologic surgery is evolving as we strive to balance the benefits of MIS with safety and oncologic outcomes.

Previous experience with MIS in various malignancies has yielded mixed results. While both prospective and retrospective studies have shown worse outcomes for early-stage cervical cancer with MIS compared to open surgery (19, 20), this is not the case for endometrial cancer (21, 22). In the context of ovarian cancer debulking, MIS has been explored more extensively in interval surgery, where the disease burden is typically lower. Small prospective feasibility studies and large retrospective reviews have demonstrated promising results. Based on these data, patients undergoing MIS IDS have improved perioperative outcomes and similar oncologic outcomes compared to patients undergoing open IDS. An ongoing prospective randomized trial (LANCE) will likely determine the future role of MIS in interval debulking surgery for ovarian cancer. The available data regarding the role of MIS in the context of primary debulking is quite limited.

Performing complex ovarian cancer debulking via MIS presents several challenges, including the need for adequate exposure in all four abdominal and pelvic quadrants and the absence of tactile feedback. To address these challenges, proper port placement, adjustments to the Trendelenburg position, and a 180-degree rotation of the robotic boom can facilitate access to all anatomical locations.

In this specific case, another surgeon initially positioned the patient and placed ports with the intention of performing a benign gynecologic procedure. The patient was positioned in Trendelenberg at a 25-degree angle, with trocars placed in the typical supraumbilical fashion. Four 8 mm robotic trocars and one 11 mm assist trocar were used, with the camera placed in the midline.

**Figure 2:**
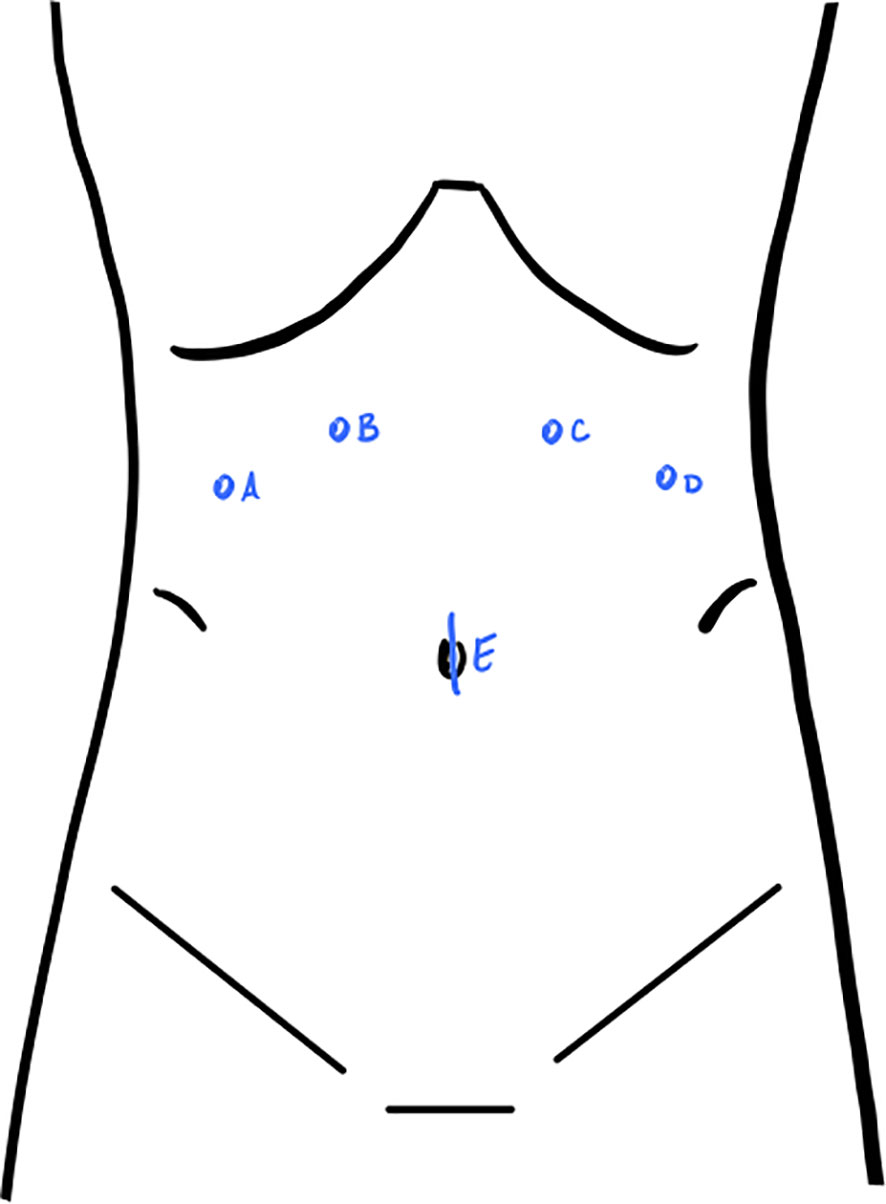
Port placement for robotic debulking. For operating in the pelvis: monopolar scissors in **(A)**, 30-degree robotic camera in **(B)**, fenestrated bipolar forceps in **(C)**, ProGrasp in **(D)**, 12mm assist trocar in **(E)**. For operating in the upper abdomen: ProGrasp in **(A)**, fenestrated bipolar forceps in **(B)**, camera in **(C)**, monopolar scissors in **(D)**.

For planned robotic debulking, our preference is to place a 12 mm trocar at the umbilicus to serve as an assist trocar throughout the case, along with two 8 mm robotic trocars in the right upper quadrant and two 8 mm robotic trocars in the left upper quadrant. The upper quadrant trocars are placed at a level halfway between the umbilicus and xiphoid (**Figure 2**). When operating in the pelvis, we position the 30-degree robotic camera in arm 3, the monopolar scissors in arm 4, the fenestrated bipolar forceps in arm 2, and the ProGrasp in arm 1. This setup allows for the visualization of enlarged pelvic pathology and the performance of pelvic and paraaortic lymphadenectomy. However, exposing the upper abdominal organs requires rotating the boom. Our practice involves undocking the robot, performing a 180-degree boom rotation to target the upper abdomen, and then redocking it after reversing the Trendelenburg position. Boom rotation enables visualization of the upper abdomen for peritoneal stripping and debulking of visible lesions. The use of a 30-degree robotic camera can be particularly helpful for exposing the posterior portion of the diaphragm and the dome of the liver. Upon rotation of the boom to operate in the upper abdomen, the camera is placed in arm 2, the monopolar scissors in arm 1, the fenestrated bipolar forceps in arm 3, and the ProGrasp in arm 4.

We chose to perform a complete pelvic and paraaortic lymph node dissection for this patient. The LION trial was published in 2019 and demonstrated no improvement in PFS or OS in patients with advanced ovarian cancer who underwent complete surgical resection of disease (23). In contrast, another prospective trial compared complete lymph node dissection to selective resection of enlarged lymph nodes and found an improvement in PFS but not OS in those who had undergone complete lymphadenectomy (24). One major difference between these trials and our patient is the surgical modality- both trials stipulated an open technique. When considering the limitation of MIS in providing tactile feedback, we chose to complete the pelvic and paraaortic lymph node dissection because, while there were not visibly enlarged lymph nodes, we could not palpate the nodal spaces. The lack of overall survival benefit in either of these trials begets the questions of the role of nodal disease in ovarian cancer mortality and the ability to salvage nodal-restricted recurrences (25). While we do acknowledge the strong evidence put forth by recent trials, we look forward to the inclusion of minimally invasive surgical debulking data before committing to the omission of lymphadenectomy in this setting.

Another potential challenge in completing primary cytoreductive surgery for advanced ovarian cancer is the technical ability to perform the additional complex procedures that are often necessary for complete cytoreduction, including bowel resection, splenectomy, and diaphragm stripping. While these procedures are technically challenging, they are not beyond the scope of minimally invasive practice and have been performed safely. Minimally invasive splenectomies, as part of complete cytoreduction for both primary and secondary cytoreduction, have been reported in case series and case reports with excellent surgical and oncologic outcomes. For instance, one study reported that six patients who underwent laparoscopic splenectomy as part of primary debulking remained disease-free at a median follow-up of 25 months (26). Techniques for minimally invasive resection of liver and diaphragm metastases have also been published (27, 28). An important consideration for multivisceral resection is placement of ports. Complete preoperative imaging assessment is especially relevant in this instance. While operation in the direct pelvis and upper abdomen (a 180-degree rotation) can be achieved with similar port placements, an additional port superior to the camera may be necessary for splenectomy. With adequate planning, minimally invasive techniques can still be utilized to resect disease in or on multiple viscera in order to achieve complete cytoreduction.

A limitation of this case report is the follow up period. We were able to achieve a good surgical outcome, but with the use of PARP inhibitors, we expect a prolonged progression free interval, especially in those with defects in intrinsic DNA repair. This patient does not have a germline or somatic BRCA mutation, but her tumor has homologous-recombination deficiency. In the PRIMA, PRIME, and ATHENA-MONO trials, the PFS was at least 22 months in a high-risk population to not reached in a population which included those with a complete cytoreduction (29–31). Continued prolonged follow up in addition to prospective randomized trials will be needed to draw conclusions on the effects of minimally invasive primary cytoreductive surgery for ovarian cancer.

## 4 Conclusion

MIS offers several benefits across different cancer types, including reduced hospital stays, minimized blood loss, and quicker initiation of adjuvant therapy. Despite the inherent challenges associated with performing multi-quadrant surgery, coupled with the absence of tactile feedback and limited exposure in specific anatomical regions, achieving complete cytoreduction via MIS in the context of primary debulking is feasible in carefully selected cases of advanced ovarian cancer.

## Data availability statement

The original contributions presented in the study are included in the article/**Supplementary Material**. Further inquiries can be directed to the corresponding author.

## Ethics statement

Written informed consent was obtained from the individual(s) for the publication of any potentially identifiable images or data included in this article.

## Author contributions

JW: Conceptualization, Writing – original draft. NG: Conceptualization, Writing – original draft. IA: Conceptualization, Supervision, Writing – review & editing.
